# Differential Impact of Tobacco Control Policies on Youth Sub-Populations

**DOI:** 10.3390/ijerph10094306

**Published:** 2013-09-12

**Authors:** John A. Tauras, Jidong Huang, Frank J. Chaloupka

**Affiliations:** 1 Department of Economics, University of Illinois at Chicago, 601 South Morgan Street, Chicago, IL 60607, USA; E-Mail: fjc@uic.edu; 2 Institute for Health Research and Policy, University of Illinois at Chicago, 601 South Morgan Street, Chicago, IL 60607, USA; E-Mail: jhuang12@uic.edu

**Keywords:** price, smoke-free air laws, youth, smoking, sub-populations

## Abstract

*Background*: While previous studies have demonstrated the efficacy of tobacco control interventions in reducing tobacco use among youth overall, there have been very few studies that examine the potential differential impact of tobacco control policies on various youth subgroups, defined by socio-economic status (SES), race/ethnicity, and gender. *Objective*: We examined the relationship between state-level cigarette prices and smoke-free air laws and youth smoking prevalence and intensity for various youth sub-populations in the United States. *Methods*: We estimated a 2-part model of cigarette demand using data from the 1991 through 2010 nationally representative surveys of 8th-, 10th-, and 12th-grade students as part of the Monitoring the Future project. *Findings*: We found that real cigarette prices are strong determinants of youth smoking. Blacks, females, Hispanics, and low-SES subpopulations are found to have a larger price response with respect to smoking prevalence than the full sample. Smoke-free air laws are found to have a negative effect on smoking prevalence for the full sample and for the male, white, and high-SES sub-populations. *Conclusions*: This research concludes that higher cigarette prices will reduce smoking prevalence rates of Blacks, Hispanics, females, and low-SES subpopulations faster than the overall youth population and other youth sub-populations. Moreover, this research concludes that smoke-free air laws will reduce smoking prevalence for the overall youth population with the largest reductions in high SES and male subpopulations.

## 1. Introduction

Despite the substantial decline in smoking prevalence over the past few decades, tobacco use continues to be the leading preventable cause of death and disease in the United States, with nearly 443,000 deaths occurring annually because of cigarette smoking and exposure to secondhand smoke [1]. Smoking and smokeless tobacco use are initiated and established primarily during adolescence. In fact, 88% of adult smokers who smoke daily report that they started smoking by the age of 18 years [2,3,4]. Currently in the U.S., nearly 4,000 people younger than 18 years of age smoke their first cigarette every day, and an estimated 1,000 youth in that age group become new daily cigarette smokers each day, translating to nearly 400,000 young people becoming daily smokers each year [3,5]. While smoking has declined among youth since the late 1990s, it is estimated that 4.3% of middle school students and 15.8% of high school students in the U.S were still smoking in 2011. In addition, the rate of decline in smoking prevalence among youth has slowed down considerably in the past nine years [6].

The World Health Organization’s (WHO) MPOWER package and CDC’s Best Practices for Comprehensive Tobacco Control Programs outlined effective, population-based strategies for preventing tobacco use among youths, which include increasing the price of tobacco products, employing media campaigns, limiting advertisements and other promotions, implementing smoke-free air policies, and reducing the availability of tobacco products for purchase by youths [7,8].

While previous studies have demonstrated the efficacy of these interventions in reducing tobacco use among youth overall, there have been limited studies that examine the potential differential impact of these policies on various youth subgroups, defined by Socio-economic status (SES), race/ethnicity, and gender. Previous literature shows that there are considerable disparities in tobacco use among various youth subgroups in the United States. For example, boys have higher rates of tobacco use than girls [6]. In addition, Native Americans/Alaska Natives and whites (especially white males) have the highest prevalence and intensity of smoking rates, and Latinos and Asians have lower prevalence and intensity of smoking rates [9]. The differential impact of these tobacco control policies on various youth sub-populations represents an important avenue to reduce tobacco use disparity among youth and young adults, as a result, it is of critical importance to understand how each youth subgroup may respond to tobacco control policies in ways that differ from other subgroups.

This study fills in this research gap and advances the literature by examining the differential responses to cigarette prices and smoke-free air policies among various American youth sub-populations defined by SES, race/ethnicity, and gender. We use the data from 1991–2010 Monitoring the Future surveys, a national representative cross-sectional survey of American middle school and high school students, to examine whether and to what extent each youth subgroup respond to changes in cigarette prices and state level smoke-free air policies and to investigate whether the responses differ depending on the membership of youth subgroups and the tobacco control policy examined.

### 1.1. Brief Literature Review

#### 1.1.1. Studies on the Differential Impact of Price on Youth Smoking

Chaloupka and Pacula were the first to examine the impact of cigarette prices, youth access laws, and smoke-free air laws on adolescent smoking prevalence by racial groups [10]. They analyzed the Monitoring the Future surveys from 1992, 1993, and 1994, and found that African Americans were twice as responsive as white adolescents, with respective estimated elasticities of demand of −1.11 and −0.64.

Gruber and Zinman used several data sources to examine the potential differential impact of cigarette prices on youth smoking among different racial groups [11]. The results of their analyses were mixed: using the (MTF) surveys, they found that neither white nor non-white younger teenagers were responsive to price changes. Younger teenagers were defined as individuals in 8th or 10th grade. On the other hand, they found older non-white teenagers to be extremely responsive to changes in cigarette prices (the total non-white price elasticity of demand is estimated to be −4.35), while older white teenagers are not affected significantly by price changes. Older teenagers were defined as those in 12th grade. Due to data limitations, they were not able to decompose the non-white racial category into different race/ethnic categories to examine what was driving the implausibly high price elasticity among the older nonwhite group. Furthermore, using the Youth Risk Behavior Surveys (YRBS), they found that both younger and older white teenagers, as well as younger black teenagers, to be unresponsive to price changes. However, they found older black teenagers to have an unreasonably high price elasticity of demand of −17.51 (prevalence elasticity of −9.26 and conditional demand elasticity of −8.25). Finally, using the Vital Statistics Natality (VSN) data, they found both younger and older black teenage mothers to be unresponsive to cigarette price changes, whereas they found both younger and older white teenage mothers to decrease their consumption of cigarettes significantly when prices increase. Younger teenagers were defined as individuals aged 13–16 years, whereas older teenagers were defined as individuals aged 17–18 years in the VSN data. The researchers attribute the mixed results and unreasonably high price elasticities for non-whites and blacks on the lack of changes in cigarette taxes (*i.e.*, a lack of within-state variation in cigarette prices) during the time that their data were collected (1991–1997).

A third paper by DeCicca and colleagues examined the impact of cigarette prices on smoking among white, black and Hispanic adolescents employing the National Educational Longitudinal Study of 1988 [12]. Unlike the previous studies that examined the determinants of average smoking and/or smoking prevalence, the study by DeCicca and colleagues examined the determinants of smoking initiation. The researchers found that price was an insignificant determinant of smoking onset between waves of data among white adolescents but had a negative effect on black and Hispanic smoking onset. The results implied that a small tax increase of 20 cents would yield a very large reduction in the probability of initiation among Hispanics and a substantial reduction in the probability of initiation for African Americans. In particular, a 20-cent increase in the price of cigarettes was found to reduce the starting hazard rate from 17.7% to 13.33% among Hispanics and from 7.8% to 6.3% for African Americans.

Using several econometric models and expanding the findings of Chaloupka and Pacula, Ding investigates the difference in price elasticity of demand between females and males, and ethnic groups of youth smokers [13]. The study finds that youth are very responsive to changes in prices, with a price elasticity of demand of −1.4. Compared to male adolescents, female adolescents were more responsive to price changes with an estimated price elasticity of demand of −3.0. In addition, the estimated price elasticities of demand for Black and Hispanic adolescents were found to be −9.1 and −2.0, respectively. As a result, Ding argues that taxation is the most effective means of deterring youth smoking initiation, specifically it is most effective for Black and Hispanic youth.

Focusing on smoking initiation and the role that cigarette taxes and prices, Nonnemaker and colleagues, find an overall small effect for gender, race and ethnicity, and gender-by-race and ethnicity [14]. The most important finding is that for black youth, both tax and prices affect their smoking initiation rates. Consistent with the results of DeCicca *et al*., when using baseline weights, Hispanic youth are also responsive to price and tax policy [12]. When stratifying for gender effects, the results were mixed; in some models females were responsive to price changes and in others they were not. A significant effect was not found for males [14].

Two studies by Cawley, Markowitz, and Tauras found higher cigarette prices to decrease the probability of smoking initiation among males but have no impact on female smoking initiation [15,16]. These gender-specific differences may help explain the mixed and inconclusive evidence of the impact of price on smoking initiation found in previous literature.

#### 1.1.2. Studies on the Differential Impact of Smoke Free Air Laws on Youth

As mentioned before, Chaloupka and Pacula also focus on the effect of smoke free air laws on smoking prevalence. Controlling only for the existence of clear indoor air laws, but not the level of enforcement, they find that smoking prevalence rates decrease for white males only [10].

Using data from the National Survey of Children’s Health, Hawkins and colleagues examine the impact cigarettes excise taxes and smoke free legislation have on tobacco use in households where there are school-age children and adolescents present [17]. They find that stronger tobacco control policies decrease parental tobacco use. It is important to note that reduced parental smoking depends on which policy is used and how the estimating model is implemented. For white families from lower income groups stronger excise taxes on cigarettes are the most effective way of reducing parental smoking [17].

A systematic review of the literature, conducted by Thomas and colleagues, found strong evidence that smoking restrictions in workplaces and public places are more effective in reducing smoking among advantaged groups. Focusing on youth, the study found that smoking restrictions in schools are a more effective way of reducing smoking for girls and younger schoolchildren [18].

To summarize, despite the significance of this topic, there are few studies to date that have examined the differential impact of tobacco control policies on various youth sub-populations. While cigarette taxes/prices, and smoke-free air policies have been shown to reduce youth smoking overall, the empirical evidence of their impact on various youth sub-populations is scarce, and the findings so far from existing literature are mixed and inconclusive.

## 2. Methods

### 2.1. Survey Data

The data for this study were extracted from the 1991 through 2010 surveys of eighth, tenth, and twelfth grade students conducted by the Institute for Social Research at the University of Michigan as part of the Monitoring the Future (MTF) Project. The modal age of eighth, tenth, and twelfth grade students is 14, 16, and 18, respectively. MTF has conducted nationally representative surveys of between 15,000 and 19,000 high school seniors each year since 1975, and of similar numbers of eighth and tenth grade students since 1991. These surveys focus on the use of tobacco, alcohol, and illicit drugs among adolescents and young adults and related beliefs about these substances. Extensive efforts are made by MTF to ensure that the data collected are informative and accurate. Students are assured of confidentiality, and all questionnaires were administered by trained interviewers.

Data on each individual’s monthly cigarette usage were used to construct two alternative dependent variables: prevalence of cigarette smoking and average monthly cigarette consumption among smokers. Prevalence of cigarette use was a dichotomous indicator equal to one for youths who indicated that they smoked cigarettes in the 30 days prior the survey; and otherwise, equal to zero. The second dependent variable was a quasi-continuous measure of the number of cigarettes smoked during the 30 days prior the survey. This variable was based on a question that asked respondents how frequently they smoked during the past 30 days. Response alternatives and their coded values were less than one per day (0.5), one to five (3), about ½ pack (10), about 1 pack (20), about 1 and ½ packs (30), and 2 packs or more (40).

Variables that were used to control for other factors thought likely to affect cigarette smoking included: the age of the respondent in years; gender (female and male-reference category); separate indicators for school grade with twelfth grade as the reference category; indicators of race/ethnicity (Black, Hispanic, other race, and White-reference category); indicators of maternal education (8th grade or less, some high school, high school graduate, some college, graduate degree, and college graduate-reference category); indicators of maternal work status (part-time, not working or missing, and full-time-reference category); indicators of living arrangement (live alone, live with father only, live with mother only, live with others that are not mom or dad, live with both mom and dad–reference category), indicators of weekly income from work and other sources ($0 < income ≤ $10; $10 < income ≤ $20; $20 < income ≤ $30; $30 < income ≤ $40; $40 < income ≤ $50; $50 < income ≤ $60; income > $60; and income = 0–reference category), and indicator for living in a rural community.

We also created mutually exclusive but all-inclusive dichotomous indicators for each year of the survey and each state in the survey. The dichotomous state indicators capture all time-invariant state-level unobserved heterogeneity and the year indicators account for overall trends in smoking over time. We employed a two-way fixed-effects regression technique in all the analyses. The fixed effects approach amounts to including a dichotomous indicator for each state (less one) and each year (less one) as explanatory variables in the models.

### 2.2. Cigarette Prices

Based on the state in which each youth’s school was located, we also merged cigarette prices with the survey data. We obtained price data from the annual Tax Burden on Tobacco. Until 1999, the Tobacco Institute published state specific cigarette prices as of November 1 of each year. Since then, Orzechowski and Walker have published the data. These prices are weighted averages for a pack of 20 cigarettes and are inclusive of state level excise taxes applied to cigarettes [19]. Because the price published was as of 1 November of each year and the surveys were conducted between February and June of each year, we created a weighted average price for the first six months of each year. To account for changes in the relative price of cigarettes over time, all cigarette prices were deflated by the national Consumer Price Index published by the Bureau of Labor Statistics (1982–1984 = 100).

### 2.3. Smoke-Free Air Laws

Using state identifiers, we merged a smoke-free air index variable with the survey data. The smoke-free air data were compiled by Gary Giovino and colleagues at the University of Buffalo for the ImpacTeen project. For each state we know whether or not smoking was banned in private worksite, restaurants, and bars at the time the respondents were being surveyed. The smoke-free air index variable is a simple tally of the number of bans each state had in the aforementioned venues and therefore the range of the smoke-free air index variable is 0–3. For example, if a state had no bans on smoking in private worksites, restaurants, and bars at the time of the survey, the index variable would take on a value of zero. If on the other hand the state has a ban on smoking in all 3 venues, the index would take on a value of three. The smoke-free air index is very similar to the index variable used by Tauras and others [20]. Table 1 provides descriptive statistics for the key independent variables used in the cigarette demand equations.

### 2.4. Statistical Methods

We used a modified two-part model of cigarette demand in which smoking prevalence and smoking intensity were estimated separately. In the first step, we used logit methods to estimate a cigarette smoking prevalence equation. In the second step, we used a generalized linear model with log link and gamma distribution to model the continuous monthly consumption measure. Both equations employed weights to account for differential sampling probabilities. The same set of independent variables was included in both equations.

Table 2 contains the smoking prevalence equations and Table 3 contains the smoking intensity equations. Model 1 in each table is for the full sample of 8th, 10th, and 12th grade students. The model specification includes the following covariates: real price of cigarettes, smoke-free air index, age, and indicators for gender, race, grade level, income, rural, maternal employment, maternal education, and current living arrangement. In addition, we include dichotomous year indicators and dichotomous state indicators as part of our two-way fixed effect approach. Finally, we also included dichotomous indicators for respondents with missing data for race, gender, living arrangement, maternal education, and income. These missing value indicators were created to prevent the loss of a large number of observations. For example, if mother’s education is unknown, each of the mother’s education variables take on a value of zero, while an additional indicator, unknown mother’s education takes on a value of one. This missing value indicator takes on a value of zero for all respondents whose mother’s education is known.

**Table 1:** Descriptive Statistics for Key Independent Variables.

Variable	Mean	Standard Deviation
Price	$3.99	$1.21
Smoke Free Air	1.82	1.87
Female	0.51	0.50
Age	15.52	1.74
Black	0.13	0.34
Hispanic	0.12	0.32
Other Race	0.11	0.31
Grade 8	0.36	0.48
Grade10	0.33	0.47
Rural	0.17	0.38
Live Alone	0.01	0.08
Father Only	0.04	0.19
Mother Only	0.18	0.39
Live with other	0.04	0.19
0 < income ≤ 10	0.29	0.45
10 < income ≤ 20	0.17	0.38
20 < income ≤ 30	0.09	0.29
30 < income ≤ 40	0.06	0.23
40 < income ≤ 50	0.05	0.22
50 < income ≤ 60	0.05	0.21
Income > 60	0.16	0.37
Mother 8th Grade	0.03	0.16
Mother Some HS	0.08	0.28
Mother HS Grad	0.26	0.44
Mother Some College	0.18	0.38
Mother Graduate School	0.12	0.33
Mother Work Part Time	0.19	0.39
Mother No Work	0.20	0.40

Model 2 of each table is a smoking demand equation for females. The covariates are identical to Model 1, however, the dichotomous indicator for gender and the missing gender indicator are omitted from the specification. Model 3 of each table is a smoking demand equation for males. The covariates are identical to Model 2. Model 4 of each table is a smoking demand equation for non-Hispanic whites. The covariates are identical to Model 1, however, the dichotomous indicators for race/ethnicity and the missing race ethnicity indicator are omitted from the specification. Model 5, Model 6, and Model 7 of each table are smoking demand equations for non-Hispanic Blacks, Hispanics, and other races (*i.e.*, not white, black or Hispanic), respectively. The covariates in Models 5–7 are identical to model 4. Model 8 of each table is a smoking demand equation for low SES individuals defined by parental education (an individual is defined as low-SES if neither parent has a college degree). The covariates are identical to Model 1, however, the dichotomous indicator for maternal education and the missing maternal education indicator are omitted from the specification.

**Table 2:** Smoking Prevalence Equations.

Variable	Model 1	Model 2	Model 3	Model 4	Model 5	Model 6	Model 7	Model 8	Model 9
	Full Sample	Females	Males	Whites	Blacks	Hispanic	Other Race	Low-SES	High-SES
Price	−0.00080 −3.98	−0.00085 −3.60	−0.00077 −3.19	−0.00067 −3.03	−0.0020 −3.83	−0.0012 −2.90	−0.00014 −0.35	−0.00076 −2.86	−0.00071 −3.27
Smoke Free Air	−0.03 −2.10	−0.02 −1.32	−0.03 −2.05	−0.02 −1.81	−0.03 −0.93	−0.03 −1.28	−0.02 −0.74	−0.01 −0.63	−0.04 −2.85
Female	0.03 3.66			0.09 9.12	−0.32 −11.18	−0.11 −4.57	0.03 1.27	0.10 7.11	0.01 0.79
Age	0.15 25.47	0.12 14.53	0.18 22.44	0.15 20.03	0.19 10.63	0.12 8.26	0.17 10.94	0.18 18.68	0.14 19.63
Black	−1.40 −63.49	−1.66 −58.83	−1.14 −44.42					−1.48 −48.68	−1.37 −54.30
Hispanic	−0.36 −18.63	−0.48 −19.45	−0.23 −10.03					−0.51 −21.43	−0.16 −6.91
Other Race	−0.24 −16.71	−0.30 −15.13	−0.19 −10.08					−0.27 −11.32	−0.21 −12.30
Grade 8	−0.00 −0.01	−0.02 −0.44	0.00 0.12	−0.12 −3.48	0.41 4.96	0.11 1.63	0.11 1.64	0.30 7.16	−0.15 −4.32
Grade10	0.12 6.15	0.17 6.99	0.07 2.74	0.08 3.72	0.24 4.35	0.12 2.43	0.21 4.44	0.31 11.47	0.04 1.74
Rural	0.03 2.74	−0.03 −1.75	0.09 6.04	−0.03 −2.06	0.38 8.80	0.08 1.99	0.32 10.11	0.02 1.06	0.03 1.84
Live Alone	0.99 25.47	1.09 15.16	0.89 18.60	0.89 14.88	1.08 12.26	0.91 10.44	0.62 7.00	1.03 16.57	0.96 17.18
Father Only	0.51 31.60	0.61 24.96	0.45 20.67	0.53 26.93	0.24 4.00	0.48 10.08	0.48 10.00	0.44 16.69	0.58 27.58
Mother Only	0.32 34.52	0.37 29.36	0.27 19.89	0.38 33.08	0.11 3.93	0.18 7.22	0.35 12.49	0.25 16.09	0.35 30.25
Live with other	0.55 32.47	0.61 26.48	0.51 20.73	0.61 25.59	0.42 10.00	0.40 8.95	0.48 10.14	0.48 19.10	0.66 26.98
0 < income ≤ 10	0.16 11.03	0.18 8.94	0.14 6.64	0.19 10.57	−0.04 −0.82	0.10 2.37	0.23 5.48	0.16 6.60	0.19 9.90
10 < income ≤ 20	0.48 31.61	0.49 23.72	0.47 20.98	0.51 27.91	0.13 2.31	0.41 9.53	0.57 12.79	0.39 15.03	0.54 27.92
20 < income ≤ 30	0.59 35.07	0.61 27.29	0.58 23.68	0.61 30.31	0.28 4.71	0.51 10.52	0.73 14.56	0.50 17.59	0.66 30.48
30 < income ≤ 40	0.72 39.94	0.75 30.48	0.72 26.99	0.75 35.02	0.27 4.00	0.66 12.32	0.83 15.42	0.60 19.01	0.81 34.66
40 < income ≤ 50	0.74 38.81	0.76 27.97	0.74 27.68	0.77 34.22	0.36 5.20	0.54 9.19	0.91 15.33	0.58 17.69	0.84 34.80
50 < income≤ 60	0.85 42.99	0.92 33.29	0.80 29.30	0.88 37.53	0.41 5.50	0.74 13.02	0.93 14.48	0.64 19.16	0.98 39.19
Income > 60	0.98 62.85	1.02 45.98	0.97 44.53	1.04 54.50	0.54 10.20	0.78 18.21	1.10 24.34	0.79 30.69	1.13 55.55
Mother 8th Grade	0.17 6.61	0.19 5.49	0.19 5.14	0.63 14.08	0.28 3.31	−0.22 −4.87	−0.06 −0.96		
Mother Some HS	0.47 31.56	0.59 29.50	0.33 15.97	0.61 32.77	0.14 2.96	0.02 0.48	0.40 10.00		
Mother HS Grad.	0.20 19.92	0.28 20.09	0.13 9.15	0.23 19.21	0.03 0.80	0.00 0.09	0.19 5.93		
Mother Some College	0.15 14.02	0.20 13.52	0.10 7.03	0.15 12.37	−0.02 −0.50	0.09 2.52	0.19 5.52		
Mother Graduate School	−0.02 −1.56	−0.02 −1.22	−0.01 −0.74	−0.03 −2.45	−0.01 −0.13	−0.00 −0.01	−0.01 −0.16		
Mother Work Part Time	−0.13 −14.69	−0.15 −11.91	−0.11 −8.58	−0.16 −15.31	0.00 0.06	−0.10 −3.56	−0.05 −1.97	−0.09 −5.46	−0.12 −11.25
Mother No Work	−0.11 −11.39	−0.13 −9.78	−0.08 −6.73	−0.12 −11.10	0.071.94	−0.15 −6.00	−0.07 −2.63	−0.04 −2.49	−0.12 −9.55
N	916,496	465,429	436,363	568,703	118,972	105,900	101,334	250,156	613,170

All equations also include and intercept, state fixed effects, dichotomous year indicators, and missing value indicators for race, gender, living arrangement, maternal education, and income. In each cell, coefficient estimates are on the top and *t*—statistics are on the bottom. The critical values for the *t*—statistics are 2.58 (2.33), 1.96 (1.64), 1.64 (1.28) at the 1, 5, and 10% significance levels, respectively, based on a two-tailed (one-tailed) test. Estimates in bold indicate significance at the 5% level for a two-tailed test.

**Table 3:** Smoking Intensity Equations.

Variable	Model 1	Model 2	Model 3	Model 4	Model 5	Model 6	Model 7	Model 8	Model 9
	Full Sample	Females	Males	Whites	Blacks	Hispanic	Other Race	Low-SES	High-SES
Price	−0.00051 −2.66	−0.00095 −3.82	−0.00014 −0.54	−0.00048 −2.37	−0.00099 −1.37	−0.00035 −0.58	−0.00050 −1.03	−0.00078 −2.75	−0.00039 −1.66
Smoke Free Air	−0.00001 −0.00	0.01 0.96	−0.01 −0.77	0.01 0.76	−0.01 −0.13	−0.01 −0.34	−0.02 −0.76	−0.01 −0.34	0.01 0.50
Female	−0.14 −14.66			−0.11 −10.57	−0.36 −8.67	−0.20 −5.51	−0.21 −7.37	−0.13 −8.71	−0.14 −11.57
Age	0.11 17.18	0.12 11.62	0.11 12.99	0.13 16.43	0.11 5.09	0.09 4.48	0.12 5.77	0.14 14.59	0.10 11.08
Black	−0.53 −20.65	−0.69 −19.05	−0.42 −12.56					−0.63 −15.17	−0.48 −14.22
Hispanic	−0.37 −17.22	−0.41 −13.48	−0.34 −11.59					−0.50 −16.50	−0.22 −7.90
Other Race	−0.02 −0.98	−0.06 −2.45	0.03 1.23					−0.10 −3.87	0.04 1.92
Grade 8	0.17 5.92	0.16 3.56	0.20 5.60	0.15 4.50	0.33 3.41	0.27 3.02	0.21 2.44	0.25 5.88	0.14 3.53
Grade10	0.12 6.44	0.13 4.89	0.12 5.15	0.12 5.82	0.09 1.38	0.14 2.34	0.16 3.09	0.21 7.83	0.08 3.29
Rural	0.06 4.63	0.05 3.23	0.06 3.51	−0.01 −0.73	0.38 6.48	0.35 6.19	0.16 4.40	−0.01 −0.68	0.08 5.12
Live Alone	0.85 24.98	0.90 14.41	0.84 19.61	0.64 14.03	0.84 9.11	0.89 9.51	0.74 8.19	0.94 16.08	0.77 15.96
Father Only	0.27 15.61	0.30 11.31	0.25 10.74	0.29 15.20	0.16 1.81	0.20 2.67	0.17 3.10	0.21 7.11	0.34 15.20
Mother Only	0.18 16.76	0.22 15.26	0.15 9.31	0.24 20.61	−0.10 −2.21	0.08 2.04	0.18 5.25	0.15 8.52	0.19 14.19
Live with other	0.34 20.14	0.34 15.40	0.32 13.20	0.38 21.16	0.03 0.43	0.27 4.74	0.27 5.16	0.28 11.56	0.41 17.03
0 < income ≤ 10	−0.20 −8.95	−0.13 −4.14	−0.28 −8.00	−0.13 −5.14	−0.37 −3.94	−0.27 −3.38	−0.31 −4.39	−0.18 −5.14	−0.22 −6.56
10 < income ≤ 20	−0.19 −7.88	−0.15 −4.60	−0.21 −5.99	−0.10 −3.86	−0.36 −3.65	−0.29 −3.56	−0.27 −3.80	−0.16 −4.46	−0.20 −5.96
20 < income ≤ 30	−0.13 −5.09	−0.06 −1.76	−0.19 −4.93	−0.05 −1.67	−0.32 −3.01	−0.27 −3.03	−0.19 −2.38	−0.12 −3.05	−0.14 −4.00
30 < income ≤ 40	−0.10 −3.81	−0.02 −0.54	−0.17 −4.32	−0.02 −0.60	−0.30 −2.50	−0.11 −1.09	−0.22 −2.73	−0.06 −1.32	−0.10 −2.90
40 < income ≤ 50	−0.04 −1.65	0.02 0.45	−0.09 −2.36	0.04 1.45	−0.39 −3.26	−0.18 −1.77	−0.19 −2.40	−0.08 −1.98	−0.03 −0.82
50 < income ≤ 60	0.01 0.28	0.06 1.54	−0.03 −0.70	0.10 3.36	−0.36 −3.05	−0.12 −1.21	−0.06 −0.69	−0.02 −0.47	0.02 0.47
Income > 60	0.23 10.19	0.24 7.74	0.22 6.60	0.31 12.82	−0.04 −0.41	0.14 1.80	0.13 1.88	0.14 4.39	0.27 8.53
Mother 8th Grade	0.36 11.25	0.34 6.98	0.39 8.82	0.58 13.99	0.62 5.28	−0.07 −1.06	0.20 2.65		
Mother Some HS	0.34 22.28	0.37 17.86	0.31 13.83	0.45 26.09	0.18 2.55	−0.02 −0.27	0.25 5.23		
Mother HS Grad.	0.16 13.20	0.18 10.09	0.16 9.21	0.20 15.12	0.04 0.67	0.01 0.26	0.10 2.57		
Mother Some College	0.06 4.29	0.06 3.20	0.06 3.25	0.09 6.13	−0.13 −2.17	−0.09 −1.46	0.03 0.68		
Mother Graduate School	−0.01 −0.29	−0.03 −1.18	0.02 0.89	−0.05 −2.66	0.12 1.49	0.09 1.23	0.05 0.98		
Mother Work Part Time	−0.08 −6.84	−0.07 −4.28	−0.09 −5.59	−0.09 −7.34	−0.02 −0.33	−0.07 −1.50	−0.05 −1.25	−0.03 −1.63	−0.08 −5.47
Mother No Work	0.02 1.54	0.02 1.27	0.01 0.92	0.01 0.49	0.06 1.18	0.02 0.55	0.01 0.34	0.05 3.04	0.05 2.98
N	182,203	91,330	87,312	132,765	10,811	17,438	16,879	58,004	115,699

All equations also include and intercept, state fixed effects, dichotomous year indicators, and missing value indicators for race, gender, living arrangement, maternal education, and income. In each cell, coefficient estimates are on the top and *t*—statistics are on the bottom. The critical values for the *t*—statistics are 2.58 (2.33), 1.96 (1.64), 1.64 (1.28) at the 1, 5, and 10% significance levels, respectively, based on a two-tailed (one-tailed) test. Estimates in bold indicate significance at the 5% level for a two-tailed test.

Finally, Model 9 of each table is a smoking demand equation for high SES individuals defined by parental education (an individual is defined as high-SES if one or both parents have a college degree). The covariates are identical to Model 8.

## 3. Results

Price is found to have a negative and significant effect on smoking prevalence for the full sample and all the subpopulations that we estimated with the exception of the other race subpopulation where price was not significant at conventional levels. Table 4 contains the estimated price elasticities of demand. The estimated prevalence price elasticity of demand for the full sample, males, and females is fairly similar at −0.259, −0.248, and −0.277, respectively. The estimated prevalence price elasticity of demand is slightly lower (in absolute value) for Whites, low-SES, and high SES youth than the full sample with elasticities of −0.205, −0.229, and −0.234, respectively. Blacks and Hispanics have the largest response to prices with estimated prevalence price elasticities of −0.718 and −0.444, respectively.

**Table 4:** Price Elasticity Estimates.

Sub Population	Prevalence Price Elasticity	Intensity Price Elasticity	Total Price elasticity
Full Sample	−0.259	−0.187	−0.446
Females	−0.277	−0.350	−0.627
Males	−0.248	ns	−0.248
White	−0.205	−0.175	−0.38
Black	−0.718	ns	−0.718
Hispanic	−0.444	ns	−0.444
Other Race	ns	ns	ns
Low SES	−0.229	−0.283	−0.512
High SES	−0.234	ns	−0.234

ns = not significant.

Price is also found to have a negative and significant effect on smoking intensity for the full sample and the following subpopulations: females, whites, and low SES. Female and low-SES youth were found to be the most price-responsive with respect to smoking intensity. The estimated price elasticity of smoking intensity for females and low-SES youth was −0.350 and −0.283, respectively. The price elasticity of smoking intensity for the full sample and for whites was smaller in absolute value with elasticities of −0.187 and −0.175, respectively.

Table 4 provides the estimated price elasticities generated from our models. Blacks, Females, and low-SES subpopulations have estimated total price elasticities that are larger in absolute value than the full sample. Hispanics have the same price response as the full sample. Males, whites, and high SES subpopulations are found to be less responsive to price than the full sample and those of other races are found not to respond to price changes with respect to either smoking prevalence or intensity.

Smoke-free air laws are found to have a negative and significant effect on smoking prevalence for the full sample and for high SES and male subpopulations. Smoke-free air laws are also found have a negative a nearly significant (7% significance level of a two-tailed test) effect on whites. Smoke-free air laws were found to be negative, but insignificant determinants of smoking prevalence among females, Blacks, Hispanics, other races, and low-SES sub-populations and to be insignificant determinants of smoking intensity in all subpopulations.

Turning to the demographic results, holding other determinants of smoking constant, females are significantly more likely to smoke than males in the full sample, white subpopulation, and in the low SES population. Females are significantly less likely to smoke than males in the black and Hispanic subpopulations. Female smokers in the full sample and in each of the subpopulations smoke significantly fewer cigarettes than their male counterparts. Older youth are significantly more likely to smoke and conditional on smoking, smoke significantly more on average than younger youth for the full sample and for each of the subpopulations. Black and Hispanic youth are significantly less likely to smoke and conditional on smoking, smoke significantly fewer cigarettes on average than white youth for the full sample and for each of the subpopulation not defined by race/ethnicity.

Youth living in rural areas are significantly more likely to smoke that youth living in urban and suburban areas for the full sample and the following subpopulations: males, blacks, Hispanics, and other race. However, for the white subpopulation, youth living in rural areas are significantly less likely to smoke than those living in urban and suburban areas. Moreover, youth smokers residing in rural areas smoke significantly more cigarettes on average than those living in urban and suburban areas for all subpopulations except whites and low SES where the coefficients are significant at conventional levels. Holding age and other covariates constant, 10th graders are significantly more likely to smoke than 12th graders. Moreover, ceteris paribus, 8th and 10th grade smokers smoke more on average than 12 grade smokers.

Youth living with both parents are significantly less likely to smoke than any other living arrangement including living alone, living with just one parent, and living with someone other than a parent. This is true for the full sample and all the subpopulations. Youth smokers living with both parents were found to smoke significantly fewer cigarettes per month than any other living arrangement, including living alone, living with just one parent, and living with someone other than a parent for the full sample and all subpopulations except the black subpopulation. Black smokers who live by themselves smoke more on average than those who live with both parents, but black smokers who with their mothers only smoke fewer cigarettes on average than black smokers who live with both parents.

Youth who generate an income from work and other sources are significantly more likely to smoke than youth who do not receive an income. This is true for all subpopulations and each income level with the exception of blacks who earn less than $10 per week, where the result is insignificant. Youth smokers who have low income levels per week typically smoke fewer cigarettes on average than those who do not receive an income. This is true for the full sample and all subpopulations for youth making less than $20 per week and for most subpopulations making less than $40 per week. However, youth smokers who have relatively high income levels (*i.e.*, incomes > $60 per week) smoke more on average than smokers who do not receive any income. This is true for the full sample and all subpopulations with the exception of blacks.

Mother’s education is a strong determinant of smoking for the full sample, females, males, and whites. In particular for these populations, youths whose mothers have less than 8th grade education, some high school, a high school degree, or some college are significantly more likely to smoke and conditional on smoking smoke at higher intensity levels than youths whose mothers have a college degree. The results with respect to Blacks, Hispanics, and other races are more mixed.

Mother’s work status is also a strong determinant of youth smoking. In particular, youth are significantly less likely to smoke if their mothers do not work or work only part time compared to youths whose mothers work full time. This is true for the full sample and all subpopulations with the exception of blacks. The results are not as strong with respect to smoking intensity. For the full sample, females, males, and whites, youth smokers whose mothers work part time smoke significantly fewer cigarettes on average than youth smokers whose mothers work full time.

## 4. Conclusions

Cigarette prices are found to be inversely related to smoking for the full sample and all subpopulations with the exception of one subpopulation—other race. The estimated total price elasticity for the full sample of youth is −0.446, implying that a 10% increase in the price of cigarettes will reduce the overall number of cigarettes consumed by youth by approximately 4.5%. Blacks, females, and low-SES subpopulations are found to have the largest overall price response. Blacks and Hispanics have the largest prevalence response with price elasticities of smoking prevalence estimated to be −0.718 and −0.444, respectively. Females have the largest intensity response with a price elastitity of intensity estimated to be −0.350.

Blacks and Hispanics having a larger prevalence price response than other subpopulations is consistent with the work of DeCicca and colleagues who examined the determinants of smoking initiation by youth of different race and ethnicities using data extracted from the 1988–1992 NELS [12]. They found price to have a sizeable negative impact on Hispanics and African American smoking initiation decisions, while having an insignificant influence on smoking initiation decisions among Whites. It is also consistent with Farrelly *et al*. who looked at adult smoking in the US [21]. Specifically, Farrelly and colleagues found African Americans to be more than twice as price-responsive as whites and Hispanics to be more than six times as price-responsive as whites.

The finding that low-SES are more price responsive than the full sample is also consistent with Farrelly and his colleagues’ findings for adults [21]. Farrelly *et al*. found low income individuals to be more than four times as responsive to changes in cigarette prices as high income individuals [21]. In particular, the authors found adults with incomes at or below the median income for the sample to have a total price elasticity of demand of −0.43 and those with incomes above the median in the sample to have a total price elasticity of demand equal to −0.10.

The finding that females are more price responsive than males is consistent with studies conducted in the United Kingdom, but for the most part, inconsistent with studies conducted in the North America. A preponderance of the studies conducted in North American countries concluded that female cigarette consumption is less responsive to changes in cigarette prices than is male consumption. Whereas, studies conducted in the UK have generally found female consumption of cigarettes to be more responsive to price changes than male consumption [22].

Finally, smoke-free air laws are found to have a negative and significant effect on smoking prevalence for the full sample and for high SES and male subpopulations. Smoke-free air laws are also found have a negative a nearly significant (7% significance level) effect on whites. Smoke-free air laws were found not to be strong determinants of smoking prevalence among females, Blacks, Hispanics, other races, and low-SES sub-populations and to be insignificant determinants of smoking intensity in all subpopulations. While the direct impact of these laws is likely limited given that youth are less likely to be frequent some of the venues covered by them, the more comprehensive laws are likely to reflect the social norms around smoking which do have some impact on youth smoking, overall and in at least some subpopulations.

The results indicate that smoking by youth subpopulations defined by gender, race/ethnicity, and socio-economic status responds differently to changes in cigarette prices and smoke-free air laws in the United States. These differential policy effects can be used to address disparities in youth smoking as part of a comprehensive tobacco control program.
